# Artificial Intelligence Challenges in the Healthcare Industry: A Systematic Review of Recent Evidence

**DOI:** 10.1049/htl2.70017

**Published:** 2025-09-02

**Authors:** Esmaeil Mehraeen, Haleh Siami, Sarah Montazeryan, Reza Molavi, Akram Feyzabadi, Iman Parvizy, Zeynab Ataei Masjedlu, Maryam Naseri Dehkalani, Sanam Mahmoudi, Alihasan Ahmadipour

**Affiliations:** ^1^ Department of Health Information Technology Khalkhal University of Medical Sciences Khalkhal Iran; ^2^ School of Medicine Islamic Azad University Tehran Iran; ^3^ Torbat Heydarieh University of Medical Sciences Torbat Heydarieh Iran; ^4^ Faculty of Medical Information and Management Shiraz University of Medical Sciences Shiraz Iran; ^5^ Boshroyeh Public Health Center Birjand University of Medical Sciences Birjand Iran; ^6^ Department of Health Information Management School of Allied Medical Sciences Tehran University of Medical Sciences Tehran Iran; ^7^ School of Management and Medical Informatics Tabriz University of Medical Sciences Tabriz Iran; ^8^ Faculty of Health Mazandaran University of Medical Sciences Sari Iran; ^9^ School of Medicine Hamadan University of Medical Sciences Hamadan Iran; ^10^ Faculty of Medical Sciences Kerman University of Medical Sciences Kerman Iran

**Keywords:** health care, learning (artificial intelligence), risk analysis

## Abstract

While AI is essential to the development of electronic health, it has challenges that, if resolved, might improve the standard of healthcare services. The purpose of this study is to classify and identify these issues in the healthcare field. The study utilised a systematic review approach, drawing data from the Scopus, Web of Science, and PubMed databases. The search results were imported into EndNote software, and experienced experts reviewed the relevant articles. The selection criteria focused on original research articles in English, published between 2019 and July 2024, that provided full text and sufficient data on AI challenges. Forty‐seven articles were included in the final analysis out of the 1453 that were identified. There were 17 categories for the obstacles, and the most common ones were technical challenges (29.8%), technological adoption (25.5%) and reliability and validity (23.4%). There are 24 categories into which the healthcare domains were divided. This article emphasises the critical importance of addressing technical challenges, enhancing reliability and validity, safeguarding patient data, and overcoming the lack of knowledge and understanding of artificial intelligence among patients and the general public to ensure the responsible and equitable implementation of AI in healthcare.

## Graphical abstract

This study conducted a systematic review of articles to classify AI challenges in healthcare into 17 categories, with the most significant being technical, technology adoption and reliability and validity challenges. The highest number of challenges was observed in the fields of psychiatry and cardiovascular medicine.

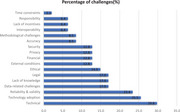

## 1 Introduction

In recent years, with the dramatic advances in information technology, artificial intelligence (AI) has emerged as one of the most advanced technologies in the healthcare industry [1, 2]. (For further clarity, all abbreviations used in this study are listed in Table B2 in Appendix B). This technology plays a significant role as a key component in the development of medicine, especially in electronic health. AI, with its ability to analyse data and provide information‐based solutions, helps improve the quality of healthcare services and heralds a significant transformation in this field. By venturing into the healthcare sector, this technology has created a fundamental evolution in diagnosing, treating, monitoring and predicting disease outbreaks. It helps in the analysis of complex medical data. These developments have improved the health of society [3].

However, this technology faces several challenges, including privacy issues, security, and ethical concerns, as well as data heterogeneity and dispersion [4, 5]. AI challenges include legal and managerial barriers. For example, the lack of trust in doctors and healthcare personnel in AI systems can reduce the adoption of this technology. The importance of public health has forced the developers of AI systems to identify and solve the challenges of this field. In addition, international organisations have also entered into this matter. For example, the World Health Organization (WHO) has initiated activities to create a global framework on AI ethics and governance, which aims to improve and distribute health services equitably [6].

The importance of AI as a transformative technology in the healthcare industry and its high ability to improve the quality of healthcare services prompted us to dedicate this article to reviewing, analysing and providing appropriate solutions in this field. Considering the wide potential of this technology, it is necessary to identify the challenges and obstacles in its implementation so that we can take advantage of its benefits in the best way. The purpose of this article is to provide a scientific analysis of the current challenges of AI in the healthcare industry. The findings from this review can aid professionals and researchers in gaining a clearer understanding of the current obstacles, designing effective strategies for integrating AI into healthcare processes, and ultimately improving the quality of healthcare services.

## 2 Materials and Methods

### 2.1 Search Strategy

The present study is a systematic review whose authors have identified the challenges of AI in healthcare based on studies conducted in Scopus, Web of Science, and PubMed scientific databases. The search strategy was performed using a combination of the terms ‘artificial intelligence,’ ‘challenges’ and ‘healthcare’ and their synonyms. Additionally, MESH was utilized to identify keywords.

### 2.2 Related Researches

The search results performed in valid databases were transferred to the Endnote resource management software, and a team of three seasoned experts in AI and healthcare meticulously reviewed the relevant articles. The selection criteria of articles included original research articles published in English and available between 2019 and July 2024, and they had to have full text. In addition, the selected articles had to provide adequate and relevant data on AI challenges in the healthcare industry. On the other hand, the exclusion criteria included duplicate articles, articles without full text, and articles that did not have sufficient and comprehensive data on the topic under review. This meticulous article selection process ensured that only high‐quality and relevant sources were included in the study.

### 2.3 Data Extraction

Following the collection of relevant articles, three experts collaborated to complete the data extraction process using a data extraction form. The form contained fields like row number, author's first and last name, publication year, study aims, type of challenge, healthcare field, and key study findings. Appendix B presents the data extraction results, offering a comprehensive organization of the articles’ key information to facilitate better analysis and comparison of the findings.

### 2.4 Quality and Risk of Bias Assessment

To optimize the quality, this review study benefits from the Preferred Reporting Items for Systematic Reviews and Meta‐Analyses (PRISMA) checklist. To minimise any probable bias risk, we utilised the Newcastle‐Ottawa Scale (NOS) risk assessment tool (Table A1 in Appendix A). It is worth mentioning that a total score of nine in three categories is calculated using this numerical bias assessment tool. These three categories include selection, comparability, and exposure/ outcome. Numerical values of four, two, and three are attributed to these categories, respectively.

## 3 Results

### 3.1 Descriptive Overview of the Included Studies

**FIGURE 1:**
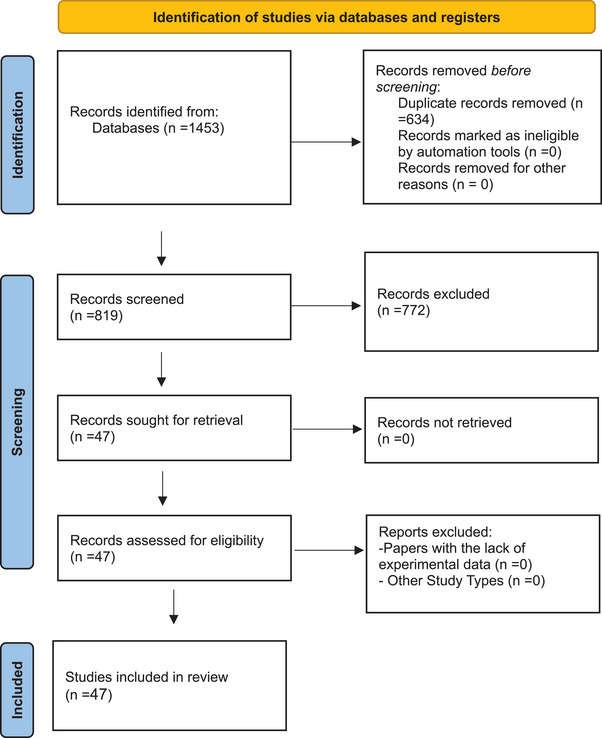
PRISMA 2020 flow diagram of the study retrieval process.

After searching PubMed, Scopus, and Web of Science databases, 1453 articles were identified. From this number, 634 duplicate articles were removed and the remaining 819 articles were screened by three expert researchers based on the title and abstract. Finally, 47 articles were included in the study based on the pre‐determined inclusion and exclusion criteria. All of the included articles were in the fair and good quality range and we did not exclude any studies after the paper quality check (Table B1 in Appendix B). The process of retrieving studies using the PRISMA tool version 2020 is reported and presented in Figure 1.

### 3.2 Identification and Analysis of AI Challenges

**FIGURE 2:**
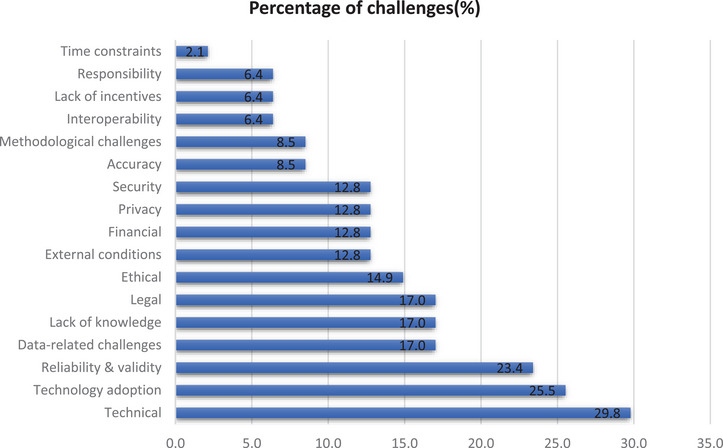
Frequency of each challenge (in percentage).

After reviewing the included studies, a total of 110 challenges were recognised and grouped into 17 categories according to their type and level of similarity. The classification and frequency of challenges are shown in Figure 2. Technical challenges, technology adoption, reliability and validity, data‐related challenges, lack of knowledge, and legal issues are the most common challenges, with 29.8%, 25.5%, 23.4%, 17.0%, 17.0%, and 17.0%, respectively.

In other findings of this paper, the challenges of AI were categorised into 24 areas of healthcare. According to this category, the fields of psychiatry and cardiovascular medicine are the most frequent, with three studies respectively. The fields of radiology, public health, geriatric medicine, digestive diseases, and biology each allocated two studies. Additionally, the fields of tuberculosis, psychology, orthopaedics, ophthalmology and dentistry, occupational therapy, neurology, nursing, musculoskeletal disorders, intensive care medicine, gynaecology, ENT, diabetes, and consultation each allocated one study. Also, 15 studies were classified in the unspecified area and labelled N/A, which indicates the need for a more precise definition of healthcare areas in future studies. These findings provide a map of the distribution of AI challenges in different subsectors of the healthcare industry and can help identify priority areas for future research.

In the studies related to each of the healthcare areas, various challenges were identified and categorised. Accordingly, the fields of psychiatry and cardiovascular medicine have the highest number of challenges, with seven and six challenges, respectively. Table 1 shows the breakdown of each healthcare area along with the frequency and type of challenges in that area. This classification can help identify weak points and research needs in each field and pave the way for developing effective strategies to improve the use of AI in healthcare.

**TABLE 1:** Healthcare domains categorised by each challenge.

Healthcare sector	Challenges																	
	Lack of knowledge	Data‐related challenges	Reliability and validity	Technology adoption	Technical	Privacy	Financial	External Conditions	Ethical	Legal	Time Constraints	Responsibility	Lack of Incentives	Interoperability	Methodological Challenges	Accuracy	Security	Frequency of Challenges(N)
Biology								✓										**1**
Biomedicine			✓															**1**
Cardiovascular medicine	✓		✓	✓	✓		✓							✓				**6**
Consultation		✓									✓							**2**
Diabetes								✓										**1**
Digestive disease		✓		✓	✓													**3**
ENT		✓		✓		✓		✓								✓		**5**
Geriatric medicine	✓					✓	✓			✓						✓		**5**
Gynecology				✓													✓	**2**
Infectious diseases					✓													**1**
Intensive care medicine		✓	✓													✓		**3**
Musculoskeletal disorders		✓	✓	✓														**3**
Tuberculosis			✓		✓													**2**
Neurology	✓																	**1**
Nursing	✓				✓													**2**
Occupational therapy									✓								✓	**2**
Ophthalmology and dentistry							✓						✓					**2**
Orthopedics									✓									**1**
Pharmacy															✓			**1**
Psychiatry	✓				✓	✓			✓	✓		✓					✓	**7**
Psychology			✓		✓											✓		**3**
Public health			✓	✓	✓				✓						✓			**5**
Radiology		✓	✓															**2**
N/A	✓	✓	✓	✓	✓	✓	✓	✓	✓	✓			✓	✓	✓		✓	**14**
**The number of challenges across all domains**	**6**	**7**	**9**	**7**	**9**	**4**	**5**	**4**	**5**	**3**	**1**	**1**	**3**	**2**	**3**	**4**	**4**	

In this systematic study, an attempt has been made to identify and investigate the main challenges of AI in the healthcare industry. After applying the inclusion and exclusion criteria, 47 articles that investigated this issue in the last 5 years were included in the study. The review of these studies led to the identification of 17 main challenges of AI in 24 different areas of healthcare. The primary difficulties highlighted in these studies were associated with the three primary domains: technical, technology acceptance and reliability and validity. Technical challenges include data quality, reliability of algorithms, and integration of AI systems with existing infrastructure. Technology acceptance also refers to cultural and organisational barriers that can affect the willingness of employees to use new technologies. The reliability and validity of AI systems are crucial as well because any defect or inaccuracy in these systems can have serious consequences for patients and health service providers. Also, other challenges, such as a lack of sufficient knowledge and data challenges, were considered in these studies. The lack of sufficient knowledge about AI and how to use it correctly is one of the main obstacles to the adoption of this technology. Data challenges are also related to issues such as access to sufficient and quality data, data preparation for use in AI systems and data privacy.

To remove these obstacles, solutions include training and transfer of experiences among specialists, removing technical obstacles and modifying work processes, forming supervisory and specialised teams, and data preparation. In addition, providing the necessary financial costs and applying health technology standards can help facilitate and accelerate the implementation of AI in the healthcare industry. These findings emphasise that there is a need for a comprehensive and multifaceted approach to effectively face the challenges of AI in this field.

Examining the fields studied in these articles showed that the most attention was paid to the fields of psychiatry and cardiovascular medicine. This can be due to the high potential of AI in medical data processing and medical imaging in these areas. For example, in the field of psychiatry, AI can help diagnose and predict mental disorders, design personalised treatment plans, and monitor the progress of patients. Also, in the field of cardiovascular medicine, AI can help in the early diagnosis of cardiovascular diseases by processing medical imaging data, such as echocardiography and CT scans and can be effective in designing treatment plans.

## 4 Discussion

The findings of this systematic review indicate that the most frequently addressed challenges in the analysed studies include technical issues and concerns related to reliability and validity in nine studies, challenges related to technology adoption and data in seven studies, and knowledge gaps in six studies. Additionally, AI‐related challenges were categorised across 24 different healthcare domains, with psychiatry and cardiovascular medicine having the highest representation at three studies each. Radiology, public health, geriatric medicine, digestive diseases, and biology were each represented by two studies, while tuberculosis, psychology, orthopaedics, ophthalmology and dentistry, occupational therapy, neurology, nursing, musculoskeletal disorders, intensive care medicine, gynaecology, ENT, diabetes, and consultation were each addressed in one study. These findings provide a comprehensive mapping of AI challenges across various subsectors of the healthcare industry and may assist in identifying priority areas for future research.

Rising healthcare costs are one of the most important challenges in this field [7]. The industry is looking for cutting‐edge ways to improve healthcare services while lowering costs to address these problems. Using AI, which has made great progress in processing vast amounts of data and mimicking human cognitive capabilities, is one viable approach [8]. However, those who utilise AI clinicians in this case are not entirely knowledgeable about or accustomed to its uses in medicine. A poor degree of awareness was indicated by the results of two studies, which found that only 23% and 27% of physicians are aware of employing AI in medicine. Nonetheless, physicians' opinions of AI in medicine were favourable in both trials [9, 10, 11]. Implementing AI applications may provide several problems, including those related to technological issues, technology adoption, dependability and validity, insufficient knowledge, data challenges, ethical dilemmas and autonomy [12].

According to our analysis, AI has gained traction in the health sector over the past 5 years, and individuals involved now recognise the significance of AI. Given the significance of patient care and health, addressing the AI‐related obstacles in this area and carrying out additional research seems imperative. The following are some of the most significant obstacles that must be wisely overcome.

### 4.1 Obstacles

#### 4.1.1 Reliability and Validity Challenges

While AI has the potential to revolutionise healthcare, it is important to proceed with caution because of the overblown hype surrounding this still‐emerging technology [13, 14]. The requirement for excellent engineering techniques and evidentiary standards for incorporating AI into current healthcare systems is one of the primary challenges. Vendors who offer stand‐alone solutions or narrow their focus to particular care areas further complicate this process [14, 15, 16, 17]. Some EHR businesses are just now starting to integrate AI features beyond rule‐based clinical decision support; thus, these systems are naturally complicated and require careful development and testing [18, 19].

#### 4.1.2 Data‐Related Challenges

Processing a significant amount of data is necessary for several AI techniques. Because of the potential ethical ramifications of collecting data, particularly patient data, it can be challenging at times. Certain classification and clustering techniques may produce extremely good results when used on relatively small amounts of data, but they may not be practical or useful [20, 21].

Before using the gathered data in AI approaches, preparation is necessary. Text data in particular needs to undergo extensive natural language processing before being used. One of the hardest problems in medical data processing is when different kinds of data, such as text, numerical, picture, and video, need to be combined using the same algorithm at times. Photographs, numerical data, 3D video sequences, medical pictures, and other forms can all be used to gather medical data. One of the challenges in healthcare data analysis is gathering reliable, accurate, and efficient data [22].

#### 4.1.3 Technical Challenges

The research and application of AI are fraught with technical difficulties. These difficulties include scalability and security concerns, the requirement for vast amounts of high‐quality data, and the difficulty of generalising beyond particular tasks [23]. Additionally, AI systems lack human‐like reasoning skills and have trouble integrating data from multiple modalities [23]. The creation of reliable learning algorithms and guaranteeing the security of these systems are important obstacles in the quest for artificial general intelligence (AGI) [24]. System scalability, adaptability and integration with current healthcare systems are further technical hurdles [25]. Interdisciplinary cooperation, improving healthcare professional education, and making investments in human resources and ongoing education are all necessary to address these problems [4].

#### 4.1.4 Adoption of Technology

Fundamental adjustments must be made to government supervision, hospital‐industry connections, and human‐AI collaboration to successfully integrate AI into healthcare systems while preserving human oversight to avoid unforeseen repercussions [26]. Adoption of AI in healthcare is also hindered by data‐related problems, including accessibility and quality [27]. Misaligned financial incentives and operational infrastructure difficulties must be addressed by the healthcare industry for AI adoption to be successful [28].

#### 4.1.5 Lack of Knowledge

One major obstacle to the application of AI in healthcare is the lack of knowledge [29]. These gaps include a lack of knowledge of AI, difficulties with explainability, and concerns about role substitution. Education, legislation, and the demonstration of AI's advantages in healthcare are all facilitating elements [30]. To tackle these issues, scholars advise creating explainability‐focused models, utilising a variety of explainable AI (XAI) techniques, and enhancing interdisciplinary cooperation [31]. Although many healthcare professionals are familiar with the fundamentals of AI, little is known about its precise uses [32]. Although AI has the potential to improve research and healthcare services, this lack of knowledge also applies to AI tools and related technologies [33].

### 4.2 Limitations and Future Studies

There are several restrictions on this study. First, since scholarly papers frequently omit specifics about AI functions because these functionalities are mostly proprietary, a few particular AI operations were unavailable. Second, several research studies on AI in healthcare, such as grey literature and reports that were not published in the databases that were chosen and examined, were left out, even with a comprehensive search strategy in place.

It is necessary to conduct further research by looking through more databases and adding more search terms. For instance, searching using terms like ‘obstacles,’ ‘barriers,’ ‘difficulties,’ and synonyms may yield results on AI issues. Our study has raised several difficulties that require more investigation for later work. What tactics, for example, can be used to handle and allay worries about professional responsibility when AI is used in treatment planning and decision assistance for healthcare? What potential effects can a lack of knowledge about AI among the general public and healthcare professionals have on the effective integration of this technology into healthcare systems? Furthermore, it is imperative to assess the advantages and possible disadvantages of integrating AI‐powered virtual health support into patient care, taking into account factors like patient involvement, accessibility, and confidence.

## 5 Conclusion

This study emphasises the essential need to address technical challenges, enhance the reliability and validity of AI systems, ensure effective protection of patient data, and tackle the widespread lack of awareness and understanding of AI among patients and the general public. These measures are crucial for promoting the responsible, ethical, and equitable integration of AI technologies into healthcare systems. While AI holds immense potential to support healthcare professionals by analysing vast amounts of medical data and assisting in informed decision‐making, thereby enhancing patient outcomes and resource efficiency, as well as revolutionising healthcare through more accurate diagnoses, personalised treatment plans, and efficient resource utilisation, it is essential to maintain realistic expectations and address pressing ethical considerations, such as transparency, fairness, and accountability, in the development, deployment, and use of AI systems. The article emphasises the need for future research to focus on developing standards for the evaluation of AI algorithms and investigating the ethical and legal implications of AI integration in the healthcare domain, ultimately paving the way for the transformative yet responsible adoption of this technology in the healthcare sector.

## Author Contributions

The conception and design of the study: **Esmaeil Mehraeen**. Acquisition of data: **Alihasan Ahmadipour**, **Haleh Siami**, **Sarah Montazeryan**, **Reza Molavi**, **Akram Feyzabadi**, **Iman Parvizy**, Zeynab Ataei Masjedlu, **Maryam Naseri** Dehkalani and **Sanam Mahmoudi**. Analysis and interpretation of data: Alihasan Ahmadipour, Haleh Siami, Sarah Montazeryan and Reza Molavi. Drafting the article: Esmaeil Mehraeen, Alihasan Ahmadipour, Haleh Siami and Sanam Mahmoudi. Revising it critically for important intellectual content: Esmaeil Mehraeen. Final approval of the version to be submitted: Esmaeil Mehraeen and Alihasan Ahmadipour.

## Conflicts of Interest

The authors declare no conflicts of interest.

## Data Availability

The authors stated that all information provided in this article could be shared.

## APPENDIX A

### A.1

**TABLE A1:** Newcastle‐Ottawa Scale (NOS) bias risk assessment of the study.

ID	First author	Selection (out of 4)	Comparability (out of 2)	Exposure/Outcome (out of 3)	Total (Out of 9)
1	N. Parchmann [1]	3	2	3	Good quality
2	I. M. Olaye [2]	3	2	3	Good quality
3	M. Nair [3]	4	2	3	Good quality
4	E. Mlodzinski [4]	3	2	3	Good quality
5	L. Petersson [5]	4	2	3	Good quality
6	E. Nichele [6]	3	2	3	Good quality
7	M. Lanne [7]	4	2	3	Good quality
8	H. Liyanage [8]	4	2	3	Good quality
9	A. K. Barwise [9]	2	2	2	Fair quality
10	G. Starke [10]	4	2	3	Good quality
11	M. Thenral [11]	4	2	3	Good quality
12	A. Zarifis [12]	4	2	3	Good quality
13	X. Zhang [13]	4	2	3	Good quality
14	M. Adil [14]	4	2	3	Good quality
15	A. Alanazi [15]	4	1	3	Good quality
16	T. Alanzi [16]	4	2	3	Good quality
17	Y. Almeida [17]	4	2	3	Good quality
18	S. Ameen [18]	2	2	2	Fair quality
19	S. Amirian [19]	4	2	3	Good quality
20	P. Apell [20]	3	2	3	Good quality
21	H. Kempt [21]	4	2	3	Good quality
22	A. Zemplényi [22]	2	2	3	Fair quality
23	A. Marotta [23]	4	2	3	Good quality
24	E. Jo [24]	4	2	3	Good quality
25	D. Li [25]	4	1	3	Good quality
26	L. P. Reis [26]	4	2	3	Good quality
27	M. Behr [27]	4	2	3	Good quality
28	A. Berti [28]	3	2	3	Good quality
29	D. Chhaperia [29]	4	2	3	Good quality
30	R. G. L. da Silva [30]	4	2	3	Good quality
31	K. Darcel [31]	4	2	3	Good quality
32	R. C. de Lima [32]	4	2	3	Good quality
33	M. Gudala [33]	4	2	3	Good quality
34	B. Z. Hameed [34]	2	2	2	Fair quality
35	N. Hendrix [35]	3	2	3	Good quality
36	B. Herman [36]	4	2	3	Good quality
37	S. James [37]	4	2	3	Good quality
38	B. Jayaneththi [38]	3	2	3	Good quality
39	S. Joshi [39]	4	2	3	Good quality
40	J. Kim [40]	3	2	3	Good quality
41	S. Boo [41]	4	2	3	Good quality
42	J. P. Grodniewicz [42]	4	2	3	Good quality
43	C. Gyldenkaerne [43]	3	2	3	Good quality
44	N. Lassau [44]	4	2	3	Good quality
45	A. Schepart [45]	4	2	3	Good quality
46	B. Schouten [46]	4	2	3	Good quality
47	M. Szymanski [47]	4	2	3	Good quality

*Note*: Good quality: 3 or 4 stars in selection domain AND 1 or 2 stars in comparability domain AND 2 or 3 stars in exposure/outcome domain; Fair quality: 2 stars in selection domain AND 1 or 2 stars in comparability domain AND 2 or 3 stars in exposure/outcome domain; Poor quality: 0 or 1 star in selection domain OR 0 stars in comparability domain OR 0 or 1 stars in exposure/outcome domain.

## APPENDIX B

### B.1

**TABLE B1:** Summary of AI challenges in healthcare.

ID	The first author [Reference]	Year	Aim of study	Type/Name of challenge	Name of health care speciality	Key findings
1	N. Parchmann [1]	2024	To determine ethical concerns, chances, and limitations of implementing an artificial intelligence‐based dashboard	Ethical, privacy and accuracy challenges	Geriatric medicine	Ethical concerns in this paper include changes in the patient‐physician relationship, impacts on social reality, redistribution of resources, fair access, privacy, accuracy, transparency, and explainability
2	I. M. Olaye [2]	2023	To describe the barriers to integrating early‐stage digital health and AI technologies in clinical practice and healthcare systems	Lack of knowledge, reliability and validity and technical	Cardiovascular medicine	Barriers in this paper include a lack of knowledge of health system technology, validation requirements, the purchase of information system technology, and the disadvantages of early‐stage digital health
3	M. Nair [3]	2023	To understand the context and stakeholder perspectives related to the future implementation of a clinical decision support system	Technical, financial and technology adoption	Cardiovascular medicine	This article shows that barriers and enablers can be the condition, the technology, cost, the adopter system, the organisation and the wider system
4	E. Mlodzinski [4]	2023	To elaborate implementation barriers and determinants for machine learning/AI algorithms	Accuracy, reliability and validity, data‐related challenges, privacy, security and interoperability	Intensive care medicine	This article shows that concerns in implementation include accuracy and reliability, data bias, privacy, security, patient safety, the doctor‐patient relationship, and workflow interruptions
5	L. Petersson [5]	2022	To explore challenges perceived by leaders in a regional Swedish healthcare setting concerning the implementation of AI in healthcare	External conditions	N/A	The challenges in this paper include external conditions, internal capacity for strategic change management, and the transformation of healthcare professions and practices
6	E. Nichele [6]	2022	To main challenges faced in identifying and responding to risk behaviours among young users seeking mental health support online	Time constraints and data‐related challenges	Consultation	The key challenges in this paper were time constraints, difficulty interpreting indirect communication from users about sensitive topics and maintaining trust
7	M. Lanne [7]	2021	To examine how artificial intelligence (AI) can be implemented in an ethically sustainable way	Ethical and security	Occupational therapy	This article shows that critical factors for trust include ensuring adequate understanding and competence in AI ethics and security, wide stakeholder participation, and transparency in AI‐based decision‐making
8	H. Liyanage [8]	2019	To form consensus about perceptions, issues, and challenges of artificial intelligence (AI) in primary healthcare	Technology adoption, data‐related challenges and external conditions	N/A	This article finds that unsupervised machine learning is currently not sufficiently mature or robust to be confidently used without checks in place
9	A. K. Barwise [9]	2024	To understand the perceived risks and benefits of using artificial intelligence (AI)	Technology adoption, data‐related challenges, external conditions, accuracy and privacy	ENT	The main challenges in this paper were integration of the AI into the workflow and the potential for alert fatigue, redundancy, perceived stigmatisation, supply–demand issues as well as transparency, accuracy, and privacy issues
10	G. Starke [10]	2023	To examine the ethical challenges of using AI‐based predictions in forensic psychiatry	Ethical and responsibility	Psychiatry	This article emphasises the importance of responsible and ethical development of AI systems
11	M. Thenral [11]	2021	To understand the perceived challenges of building, deploying, and using AI‐enabled telepsychiatry for clinical practice in India	Ethical, legal, responsibility, legal, privacy, security, responsibility and lack of knowledge	Psychiatry	The major concerns reported in this paper were ethical, legal, accountability, and regulatory issues, privacy, confidentiality, security, hacking, data ownership, and a lack of specific guidelines for AI‐enabled telepsychiatry
12	A. Zarifis [12]	2021	To explore whether trust and privacy concerns are barriers to the adoption of AI in health insurance	Reliability and validity and privacy	N/A	This article stated that trust was significantly lower when AI was visible/explicitly revealed to consumers during the health insurance purchasing process, compared to when AI use was not visible or explicitly stated
13	X. Zhang [13]	2024	To explore an effective method to identify and solve the psychological development problems of left‐behind children	Accuracy, technical and reliability and validity	Psychology	The main challenges in this paper were accurate data collection and preprocessing of ECG signals, effective feature selection from complex physiological data and data validity
14	M. Adil [14]	2024	The aim of this text is to examine IoT in the healthcare sector, focusing on AI‐enabled EEC technology. This study addresses the identification of unresolved security challenges in the healthcare field.	Security	N/A	The main findings of this article suggest that the discussed research directions could be useful for securing the EEC paradigm used in HC‐IoT applications, and it proposes the design of a foolproof security platform to maintain the trust of stakeholders.
15	A. Alanazi [15]	2023	This study seeks to explore the current and potential applications of AI while also investigating the associated challenges.	Technical and legal	N/A	Artificial intelligence has the potential to transform healthcare through its integration with EHRs and other existing technologies, but challenges must be addressed before this potential can be realised. The development and testing of complex AI‐powered systems require accuracy and reliability in treatment decision‐making, adherence to medico‐legal obligations, and assurance of equitable distribution of benefits.
16	T. Alanzi [16]	2023	This study aims to explore the factors associated with artificial intelligence (AI) and patient autonomy in obesity treatment decision‐making.	Ethical and technical	Public health	The study highlights the need for clear ethical guidelines, regular audits of AI algorithms, patient education, and involvement in AI‐related decision‐making. These measures are essential to ensure that AI technologies in healthcare comply with ethical principles, protect patient autonomy, and foster trust.
17	Y. Almeida [17]	2020	This paper introduces the AI‐Rehab framework for BRaNT and discusses the challenge of profiling with limited data. It also presents alternative AI solutions that may be applicable once sufficient data becomes available.	Lack of knowledge	Neurology	The BRaNT project aims to create an innovative cognitive rehabilitation tool that allows healthcare professionals to monitor and adapt treatments at home. It integrates artificial intelligence (AI) to enhance the existing Task Generator (TG) tool.
18	S. Ameen [18]	2022	This article highlights the need for nuanced and balanced approaches in the deployment and evaluation of AI systems in colorectal cancer (CRC) to enhance their benefits and mitigate negative unintended consequences in clinical decision‐making and patient care.	Data‐related challenges, technology adoption and technical	Digestive disease	The authors recommend developing a robust mixed methods framework for auditing and evaluating AI systems before clinical integration.
19	S. Amirian [19]	2023	In this contribution, an attempt was made to outline several key challenges and opportunities of XAI in orthopaedics	Ethical	Orthopaedics	The text highlights that successfully implementing explainable AI (XAI) in orthopaedics requires a collaborative, user‐centric approach, comprehensive training, attention to ethical issues, and the demonstration of value
20	P. Apell [20]	2023	This study evaluates the performance of the innovation system and identifies system‐blocking mechanisms for AI healthcare technology innovations in the life sciences industry.	External conditions	Biology	This study shows that to improve innovation system performance, policy interventions intended to increase available resources and to formulate vision and mission statements to improve healthcare with AI technology innovations may be encouraged.
21	H. Kempt [21]	2022	This paper explores using AI‐DSS for second opinions in medical diagnostics, addressing epistemological and ethical peer‐disagreement, and proposes a rule to overcome related challenges	Ethical and legal	N/A	This paper shows that the role of the physician in the diagnostic process is a priority, but the development of AI‐DSS can potentially replace physicians in various fields
22	A. Zemplényi [22]	2022	To provide recommendations for integrating AI into HTA processes, focusing on CEE countries, and explore using AI to generate evidence for decision‐making	Data‐related challenges, technical, legal, technology adoption and methodological challenges	N/A	This article finds that AI's potential in HTA for evidence generation and evaluation is underutilised. Raising awareness and securing political commitment are needed to improve regulations, infrastructure, and knowledge for better AI integration in HTA
23	A. Marotta [23]	2022	To provide an analysis of the role of AI in affecting women's healthcare and an overview of the liability implications caused by AI mistakes	Security and technology adoption	Gynaecology	This article finds that technical professionals must ensure data security, apply explainability to make AI understandable, and use traceability to track decision attributes
24	E. Jo [24]	2023	To examine the case of CareCall, an open‐domain chatbot that aims to support socially isolated individuals via check‐up phone calls and monitoring by teleoperators	Reliability and validity, technology adoption, methodological challenges and technical	Public health	This article finds that implementing long‐term memory can enhance emotional support in LLM‐driven chatbots. Better resources and processes are needed to balance open‐domain and task‐oriented chatbots. Scaling chatbots for diverse public health needs requires mechanisms for target populations and care professionals to contribute to dialogue datasets
25	D. Li [25]	2022	To develop an artificial intelligence (AI) system that could provide uncertainty estimates for its predictions and use reliability intervals (RIs) as references to help health professionals make more informed decisions	Reliability and validity	Radiology	This article finds that incorporating uncertainty estimation with Bayesian neural networks allowed the AI system to alert users to unreliable predictions, helping health professionals make better decisions. This approach improved performance and trust compared to using the AI system alone
26	L. P. Reis [26]	2023	To evaluate the perception of ophthalmologists and dentists in the use of AI technological innovations in relation to the benefits for patient care, as well as the challenges for its implementation and adoption.	Lack of incentives and financial	Ophthalmology and dentistry	The study found that AI can diagnose quickly but cannot replace professionals. AI aids diagnosis and data reliability, but patient‐professional relationships are crucial. Challenges include a lack of insurance incentives and high implementation costs. Limitations are the small, regional sample
27	M. Behr [27]	2023	To focus on conceptual and methodological challenges for the application of AI to RWD, to ground the co‐occurring hype of RWD and AI in the realities of practical applications for pharmaceutical R&D.	Methodological challenges	Pharmacy	This article finds that reliable RWD analyses are crucial for developing precision medicines. Conventional statistical models are often suitable, but AI can uncover complex unknowns. Future innovations will address AI's current limitations
28	A. Berti [28]	2023	This paper outlines the AI research in health and well‐being by a multidisciplinary team at Italy's National Research Council, highlighting both potential and real‐world challenges	Legal, technology adoption, privacy and technical	N/A	This paper finds that AI can improve health systems, but future research should involve healthcare professionals, comply with regulations, enhance transparency and privacy, integrate with existing tech, explain decisions, and establish evaluation metrics and guidelines
29	D. Chhaperia [29]	2024	This research explores the challenges and aspirations of India and similar nations in using AI for healthcare, emphasising the need for government action to make AI technologies affordable and accessible, reducing mortality rates and offering hope to millions	Financial, interoperability andlegal	N/A	This article finds that Governments must make AI technologies affordable and accessible, fostering innovation, partnerships, and policies to ensure widespread reach
30	R. G. L. da Silva [30]	2024	This article aims to explore the advancement of artificial intelligence in biomedical research and health innovation, highlighting its implications, challenges and opportunities in emerging economies	Reliability and validity	Biomedicine	This article finds that analysing AI's social and political implications in health can strengthen global biomedical knowledge, promoting trustworthiness and equitable access to address global health issues
31	K. Darcel [31]	2023	To identify the barriers that patients, providers, and health leaders perceive in relation to implementing AI in primary care and strategies to overcome them	Reliability and validity	N/A	The most important finding was that Participants were hopeful about AI but saw trust as a major barrier to adoption. Four key themes emerged, with strategies like participatory co‐design proposed to overcome these barriers. These findings highlight concerns about AI implementation in primary care
32	R. C. de Lima [32]	2024	To explore the increasing impact of AI in these scenarios of biological threats, considering not only its positive contributions but also the emerging challenges and risks that this technological advancement might unleash	External conditions	Biology	This article finds that a collaborative, multidisciplinary approach is essential for genetic enhancement and biological agents. AI's role in biological threats requires innovation, ethical consideration, and careful governance to mitigate risks
33	M. Gudala [33]	2022	To assess the benefits, barriers, and information needs that can be provided by an artificial intelligence–powered medication information voice chatbot for older adults	Lack of knowledge and financial	Geriatric medicine	This article shows that a voice‐based medication chatbot aids vision and dexterity issues, enhances knowledge and adherence, and supports health. But barriers are tech familiarity and cost. Needs are usability, reminders, and side effects information
34	B. Z. Hameed [34]	2023	To identify factors influencing healthcare providers' intentions to adopt artificial intelligence in healthcare (AIH)	Technology adoption	N/A	This article stated that performance expectancy, effort expectancy, and initial trust boost healthcare providers’ intentions to use AIH. Personal innovativeness, task complexity, and technology traits affect effort expectancy for AIH adoption
35	N. Hendrix [35]	2022	To highlight aspects of artificial intelligence (AI) that challenge traditional health technology assessment methods and identify opportunities for health economists to evaluate clinical AI	Technical and financial	N/A	In this paper, challenges include AI's generalizability across settings, integration into clinical workflows, ability to improve over time, impacts on clinician productivity, and cost uncertainties
36	B. Herman [36]	2022	To elaborate on the drug‐resistant tuberculosis (DR‐TB) problem and assess the impact of implementing an artificial intelligence application on rifampicin‐resistant tuberculosis (RR‐TB) screening	Technical and reliability and Validity	Tuberculosis	This article stated that there are concerns about their AI application's screening performance and the reliability of data collection for input parameters
37	S. James [37]	2023	To examine how young adults with Type 1 Diabetes transitioning to university adapt their self‐management practices, in order to understand how AI‐enhanced technologies could provide opportunities and challenges for supporting care during this life transition	External conditions	Diabetes	In this paper, AI and closed‐loop systems show promise, but complex social and contextual factors may need human‐centred design approaches to support young adults during this transition
38	B. Jayaneththi [38]	2023	To discuss the importance of adopting AI in healthcare, the importance of data security in AI‐enabled Medical Device Software (MDS), and the data security challenges that AI has brought to the healthcare industry	Security	N/A	The authors conclude that addressing these six challenges is crucial for AI‐enabled MDS trustworthiness: preventing data breaches, adversarial attacks, cyberattacks, insider threats, a lack of skilled staff, and the complexity of security standards
39	S. Joshi [39]	2022	To identify and analyse the implementation barriers of artificial intelligence (AI) in public healthcare systems in developing countries	Lack of knowledge, legal, methodological challenges and lack of incentives	N/A	This article stated that key barriers to AI in public healthcare are low AI awareness, lack of legal knowledge, poor future planning, and low commitment from top management
40	J. Kim [40]	2024	To investigate the efficacy of using cough sounds as a diagnostic tool for COVID‐19, and AI model applicability for new variants	Technical	Infectious diseases	AI models trained on early pandemic data may not remain effective as the virus evolves
41	S. Boo [41]	2023	To explore nurses’ views on the facilitators and barriers of implementing an AI/IoT‐based healthcare pilot project for older adults in South Korea	Technical and lack of knowledge	Nursing	This article stated that technical challenges and disparities in digital literacy among older adults pose significant barriers to implementation
42	J. P. Grodniewicz [42]	2023	To investigate and outline the major challenges in developing fully‐fledged AI‐based psychotherapy	Lack of knowledge and technical	Psychiatry	Challenges stated in this paper are limited understanding of effective psychotherapy, unclear if AI can build therapeutic relationships, and current AI's narrow scope for conducting psychotherapy
43	C. Gyldenkaerne [43]	2020	To examine challenges of applying AI to EHR data, focusing on tensions between clinicians’ primary use and AI's secondary use	Technology adoption	Digestive disease	The most important finding was that applying AI to EHR data introduced a conflict between primary use (by clinicians for patient care) and secondary use (for AI analysis)
44	N. Lassau [44]	2020	To organise three AI data challenges using CT and MRI imaging to address public health issues, build large multicentre databases, and include 3D information and prognostic questions	Data‐related challenges	Radiology	The challenges involved complex 3D imaging data and prognostic questions, representing an increase in difficulty from previous challenges
45	A. Schepart [45]	2023	To evaluate current awareness, perceptions, and clinical use of AI‐enabled digital health tools for patients with cardiovascular disease, and challenges to adoption	Lack of knowledge, technology adoption, financial, interoperability and reliability and validity	Cardiovascular medicine	Five major challenges identified in this paper are limited knowledge of AI among cardiologists, insufficient usability and integration into clinical workflows, cost constraints, poor electronic health record interoperability, lack of trust in AI tools
46	B. Schouten [46]	2022	To identify barriers and facilitators to AI implementation in clinical practice, and find general insights that could be applicable to a wide variety of AI tool implementations in medical practice	Lack of incentives	N/A	There is insufficient tension for change to facilitate widespread implementation and adoption
47	M. Szymanski [47]	2022	Evaluate different explanation types (textual, visual, hybrid) for chronic pain management recommendations and study how personal traits (need for cognition, ease‐of‐satisfaction) influence user perception	Technology adoption, reliability and validity and data‐related challenges	Musculoskeletal disorders	Challenges stated in this paper are: limited adoption by lay users, risk of over‐reliance, addressing cognitive biases, balancing information completeness and conciseness, and ensuring true interpretability

**TABLE B2:** Abbreviations and their expansions.

Row	Abbreviation	Expansion
1	3D	Three‐dimensional
2	AI	Artificial intelligence
3	AIH	Artificial intelligence in healthcare
4	CEE	Central and eastern Europe
5	CRC	Colorectal cancer
6	DR‐TB	Drug‐resistant‐tuberculosis
7	DSS	Decision support system
8	ECG	Electrocardiogram
9	EEC	Electroencephalogram
10	EHRs	Electronic health records
11	ENT	Ear nose throat
12	HC‐IoT	Healthcare–Internet of Things
13	HTA	Health technology assessment
14	LLM	Large language model
15	MDS	Medical device software
16	N/A	Not available
17	R&D	Research and development
18	RIs	Reliability intervals
19	RWD	Real‐world data
20	TG	Task generator
21	XAI	Explainable AI
